# *Asclepias Syriaca* (Common Milkweed) flowering date shift in response to climate change

**DOI:** 10.1038/s41598-018-36152-2

**Published:** 2018-12-13

**Authors:** Aaron F. Howard

**Affiliations:** Biology Department, Franklin & Marshall College, Lancaster, 17604 USA

## Abstract

The consequences of altered flowering dates due to climate change can be severe, especially for plants that rely on coordinated flower and pollinator emergence for reproduction. The plant *Asclepias syriaca* (Common Milkweed) relies on pollinators for movement of its pollen and evidence suggests that it has recently been declining. Given these factors and this plant’s importance as a host species for the declining *Danaus plexippus* (Monarch Butterfly), it is critical to determine if its flowering is being modified by climate change. As a first step to answering this question I quantified the relationship between climate and flowering date for *A. syriaca* using data from the USA National Phenology Network repository and the National Oceanic and Atmospheric Administration. I found that temperatures were higher than they had been historically (1895–2010) and mean flowering dates occurred earlier with higher temperatures. Additionally, there is a significant negative interactive effect of temperature and year on flowering date indicating that from 2011 through 2016 higher temperatures are correlated with increasingly earlier flowering dates. The change in flowering appears to be symmetrical in regards to the flowering time distribution, in that along with the mean, both maximum and minimum flowering dates are occurring earlier, as well. There is no evidence that earlier flowering is due to earlier initial growth or results in later fruit ripening. Consequences of this shift in flowering can only be speculated upon at this point, but due to the ecological importance of *A. syriaca* and its susceptibility to phenological mismatch, they should be considered when developing conservation plans for *A. syriaca* and the organisms for which it is a host.

## Introduction

The response of organisms, spanning taxa and habitat type, to climate change is a well documented phenomenon^1^. The ranges of mobile organisms are changing and the timing of life history events for all organism types are shifting in the direction predicted by the global warming associated with climate change^2–5^. For example, many plant species’ flowering times have occurred earlier in the year, which has many negative consequences for the plants including phenological mismatches between their blooming periods and their pollinators’ flight periods^6,7^. Reduced visitation by pollinators can cause decreased fruit production through pollen limitation or exacerbate pre-existing pollen limitation^6,7^.

*Asclepias syriaca* (Common Milkweed) is a plant with a highly specialized pollination system that requires insect visitors to transport pollen, making it susceptible to pollen limitation^8^ and reduced fruit production if its flower period shifts. Additionally, recent studies have indicated that the Common Milkweed is declining in the USA by proportions ranging from approximately 50 through 90%, depending on geographic area and habitat^9–11^. Its decline is especially concerning because it may be contributing to the decline of *Danaus plexippus* (Monarch Butterfly)^10,12^, which relies on *A. syriaca* as a host plant^13^. Given *A. syriaca*’s potential susceptibility to the negative consequences of phenological shift, recent decline, and importance as a host plant, it is critical to this plants future conservation that its potential response to climate change is investigated. As a first step toward that goal, I used phenological and climate data from the USA National Phenology Network (USA-NPN)^14^ along with climate data from the National Oceanic and Atmospheric Administration (NOAA)^15^ to examine the relationship between *A. syriaca* flowering date and climate (temperature and precipitation) from 2011 through 2016.

## Results

**Figure 1:**
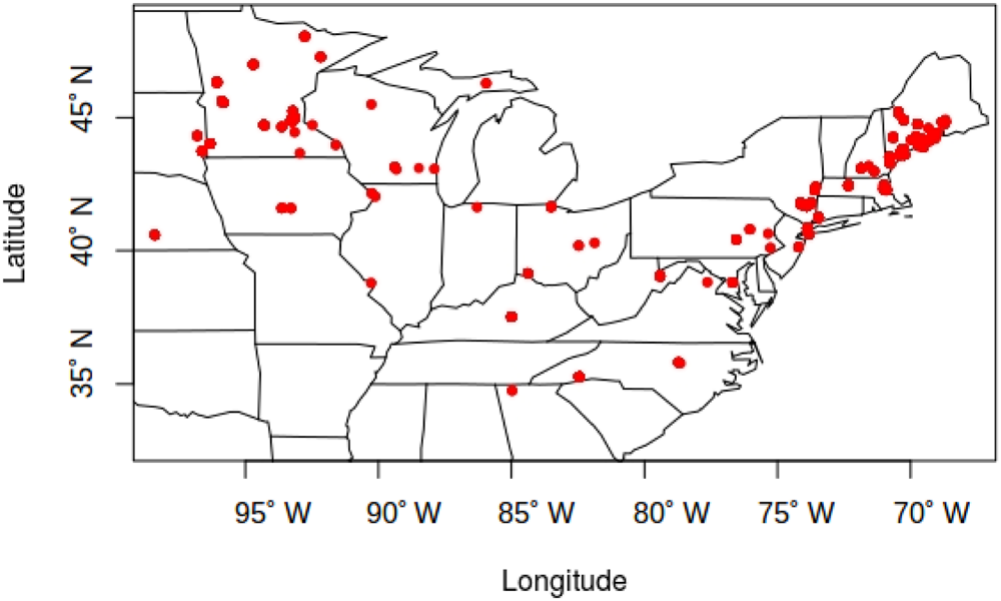
Location of *Asclepias syriaca* plants included in the analysis (red dots). I retrieved the location data (latitude and longitude) from the USA National Phenology Network data repository^14^. The map was produced in the R Statistical Program^35^ using the maps package^38^ and data from the US Census Bureau^39^.

**Figure 2:**
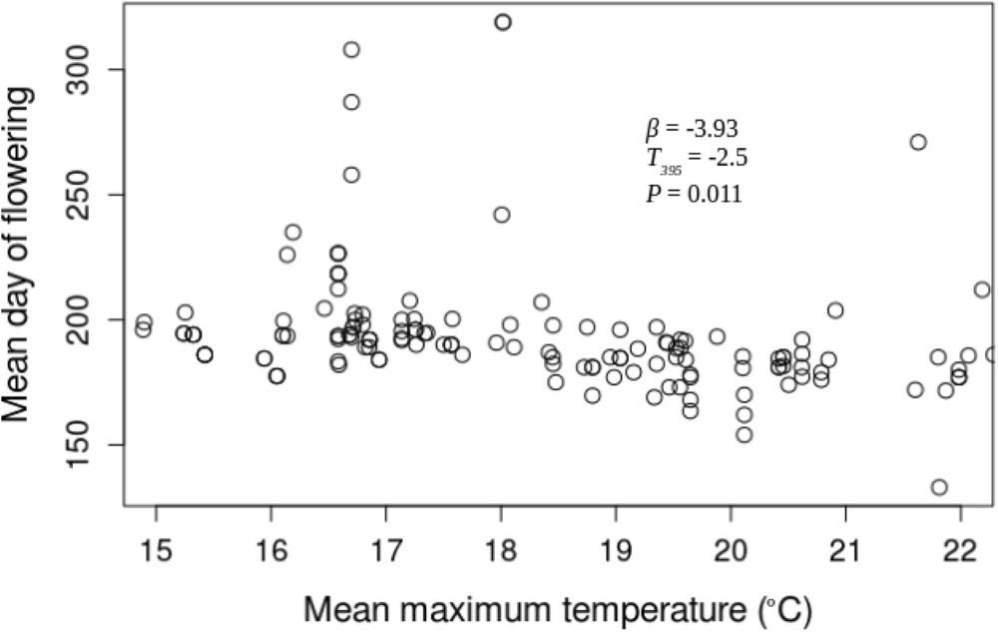
Mean flowering Julian date of *Asclepias syriaca* versus mean growing season maximum temperature (°C) by plant from 2011 through 2016. Statistics are calculated from a repeated measures linear regression controlling for latitude, elevation, precipitation, and the collection of data from individual plants during multiple years. The phenological and temperature data were from the USA National Phenology Network data repository^14^.

**Figure 3:**
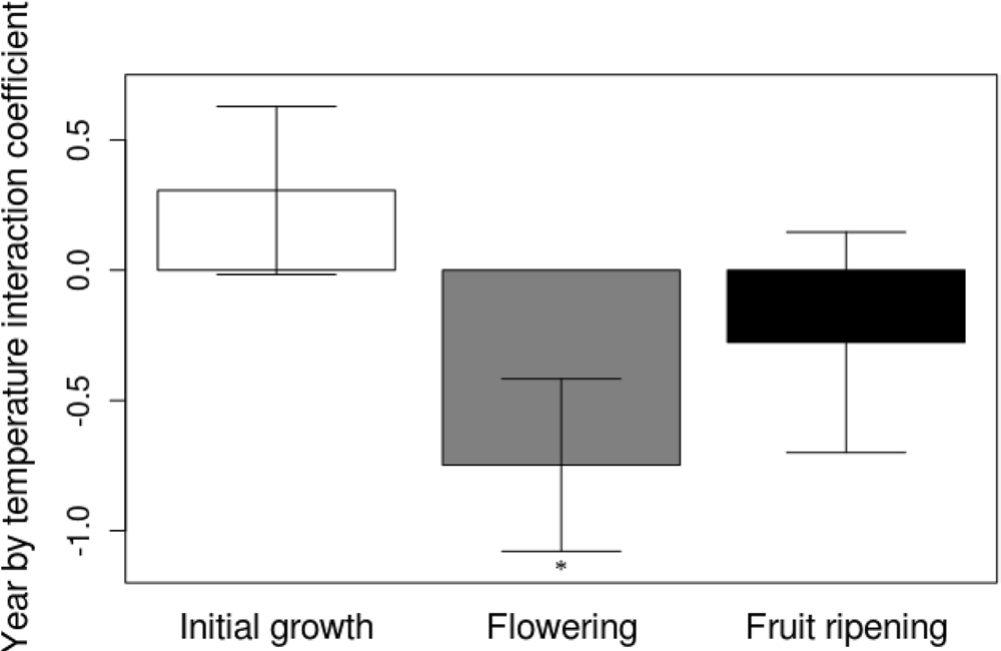
Year by growing season maximum temperature interaction coefficients (±SE) for the mean Julian date of initial growth, flowering, and fruit ripening of *Asclepias syriaca*. Coefficients and SEs are calculated from a repeated measures linear regression controlling for latitude, elevation, precipitation, and the collection of data from individual plants during multiple years. Statistical significant (p < 0.05) is indicated by an asterisk. The phenological and temperature data were from the USA National Phenology Network data repository^14^.

**Figure 4:**
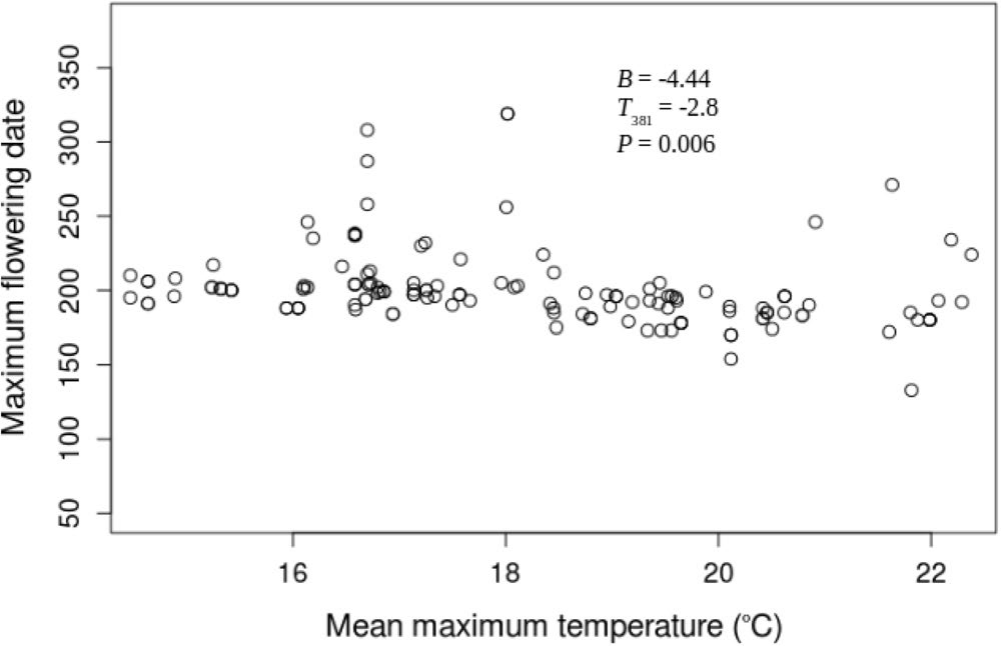
Maximum (last) flowering Julian date of *Asclepias syriaca* versus growing season mean temperature (°C) by plant from 2011 through 2016. Statistics are calculated from a repeated measures linear regression controlling for latitude, elevation, precipitation, and the collection of data from individual plants during multiple years. The phenological and temperature data were from the USA National Phenology Network data repository^14^.

**Figure 5:**
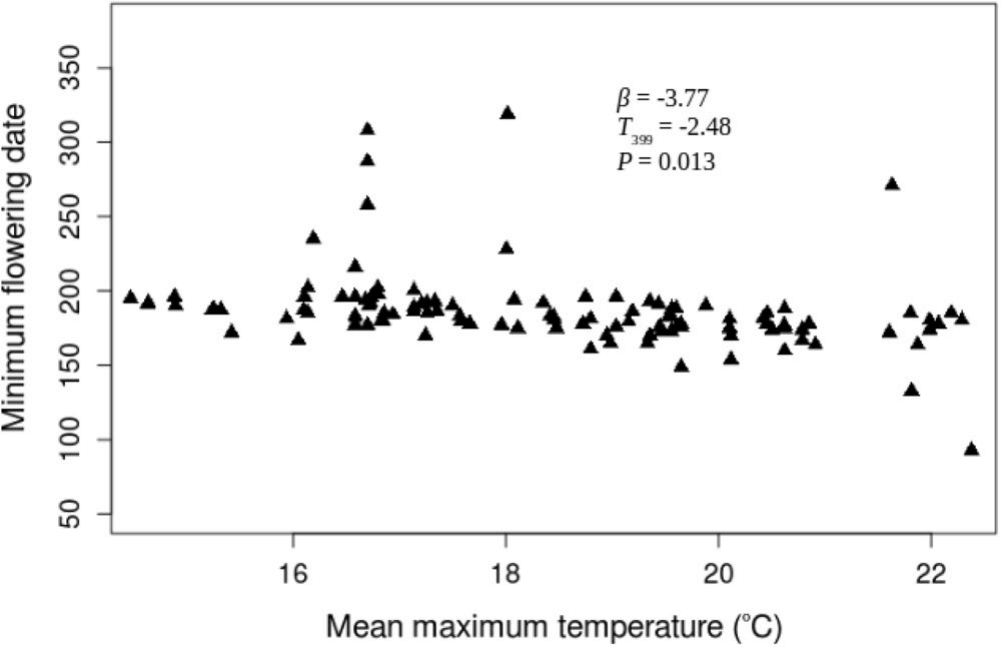
Minimum (first) flowering Julian date of *Asclepias syriaca* versus growing season mean temperature (°C) by plant from 2011 through 2016. Statistics are calculated from a repeated measures linear regression controlling for latitude, elevation, and the collection of data from individual plants during multiple years. The phenological and temperature data were from the USA National Phenology Network data repository^14^.

The USA-NPN repository includes a total of 39428 phenological status measurements for *A. syriaca*. 7158 of those status measurements are “yes” measurements indicating the presence of one phenophases: initial growth, flowering, or fruit ripening from 220 *A. syriaca* plants between the years 2011 and 2016. For this analysis, I only utilized the 1377 “yes” measurements that followed “no” measurements within a given year and were within the latitude range for plants observed in 2011. The dataset includes plants from 23 states and latitude and longitude ranges of 34.75° N–48.05° N and 68.70° W–98.4° W, respectively (Fig. 1). In the 23 states, maximum growing season temperature is significantly greater during the study period (2011–2016 mean = 19.17 °C ± 1.04) than historically (1895–2010 mean = 18.38 °C ± 0.68, *t*_*4.7*_ = 1.837, *P* = 0.048). Growing season precipitation during the study period is not different from historical values. None of the three phenophases correlated with growing season precipitation through time, and neither initial growth nor fruit ripening changed with maximum growing season temperature. However, mean flowering date decreased with temperature (*β* = −3.93, *t*_395_ = −2.5 *P* = 0.011; Fig. 2), and that relationship increased from 2011 through 2016, as indicated by the significant, negative temperature by year interaction term (*β* = −0.748, *t*_387_ = −2.261 *P* = 0.024; Fig. 3). This relationship appears to be driven by a shift in the entire flowering period because both the end (*β* = −4.44, *t*_381_ = −2.8, *P* = 0.006; Fig. 4) and beginning of flowering occur earlier as temperature increases (*β* = −3.77, *t*_28_ = −2.48 *P* = 0.013; Fig. 5). There was no relationship between flowering and either initial growth or fruit ripening (*r* = −0.06, *P* = 0.69 and *r* = −0.21, *P* = 0.15).

## Discussion

During a 6 year time period (2011–2016) *A. syriaca*’s mean flowering dates have shifted with temperature, where with each degree of temperature increase the mean flowering date has decreased by 3.93 days (Fig. 2). More importantly, temperatures in the 2011–2016 time span are significantly higher than they were historically, and flowering date is likely occurring earlier in response to temperature during that time period (Figs 2 and 3). These results indicate that this plant may be responding as predicted to global warming. Flowering did not correlate with initial growth or fruit ripening at the individual plant level, suggesting that temperature may be directly influencing flowering date. Moreover, the change in flowering seems to be driven by a shift in the entire flowering period because mean, maximum (last), and minimum (first) flowering dates are occurring earlier as temperatures increase (Figs 2–5).

The shift in the mean, minimum, and maximum flowering dates indicate that temperature is likely influencing when *A. syriaca* is producing flowers, and not some other aspect of flowering phenology such as flower longevity. The correlation between temperature and maximum flowering date is larger than the correlation between temperature and minimum flowering date, but not significantly so (Figs 4 and 5). However, given more sampling or nuanced analyses this difference may become significant because many studies have found that increasing temperature significantly decreases the amount of time that flowers persist^16–18^. In other words, temperature may not influence just when *A. syriaca* begins producing flowers, but how long the flowers bloom. The flowers on *A. syriaca* form as a series of umbellate cymes (inflorescences) that bloom chronologically along stems in a basal to apical manner^19,20^. If each cyme’s longevity is reduced, then the collective duration of flowering across the season could also be condensed, explaining why maximum (last) flowering dates occur relatively earlier, though not significantly so, than minimum (first) dates.

Changes in flowering time may negatively influence *A. syriaca*’s reproduction by disrupting the match between the timing of its flowering and its pollinators flight seasons. I am not aware of any studies that have investigated the influence of climate change on *A. syriaca* flowering time or its interactions with pollinators, but there are many recently documented plant-pollinator phenological mismatches^6,7,21^, indicating it could be an issue for this species. The negative consequences of a phenological mismatch for *A. syriaca* could be significant, especially in pollen limited populations, because of its derived pollinium-pollination system that requires vectors for pollen dispersal^8^. However, it is also possible mismatches will not occur because the *A. syriaca* flowering times and pollinator flight seasons could change in kind with increasing temperatures, as has been observed with other plant-pollinator interactions^22^. Also, given that *A. syriaca* is pollinated by a wide array of insects including many native bees, lepidopterans (moths and butterflies), and the non-native *Apis Mellifera* (Western Honey Bee), if mismatches do occur with some pollinator species others may carry out the required pollen movement and minimize the negative consequences of mismatches^23^.

An important but difficult question to ask, given the results of this study, is whether observed changes in phenology are the result of evolutionary responses or phenotypic plasticity^24,25^. The changes in *A. syriaca* flowering during this short period of time mirror the temperature patterns across the US indicating that they are the result of plasticity and not a genetic response. However, given the relatively long term increase in temperature and the forecasting of a continued increase in the foreseeable future^1^, genetic responses to climate change that have occurred in other plants may occur in *A. syriaca*, as well^25,26^. What, exactly, these evolutionary responses may be is difficult to say, as predictions are generally broad ranging, but they may come in the form of modified responses to seasonal variation or tolerance of long term changes to temperature^26^.

The data for this study were collected by a wide array of individuals, including scientists and non-scientists. The drawbacks of untrained and unsupervised individuals collecting data are clear and established^27,28^, and the possibilities of biases in the data cannot be denied. However, many steps have been taken by projects like the USA-NPN to minimize the drawbacks, such as providing methods that are accessible to non-scientists, as well as, organized, easy to use data collection sheets^14^. And the benefits to citizen science data outweigh the costs^27,28^. It would have been logistically and fiscally very difficult to collect data on *A. syriaca* plants from 23 states during the previous six years as an individual or with a small research team, and the dataset continues to grow, allowing us to determine if this trend in flowering time continues as temperatures continue to rise^1^.

One potential drawback of using citizen scientist data that is important to this analysis is a potential lack of precision and continuity of data collection. For example, individual citizens may not observe plants throughout a growing season or even from start to finish of an individual phenophase. This could lead to significant bias in these data and my interpretation of them. I attempted to mitigate this bias of incomplete sampling by only using “yes” status measurements for a phenophase within a given year that were preceded by “no” measurements. In addition, using these more complete sets of measurements may also indicate that they were collected by citizen scientist that are more engaged and invested in the goals of USA-NPN and, therefore, collect more precise data.

Lack of precision in data collection could result in biased outcomes such as the observed absence of correlations between initial growth, fruit ripening, and flowering time. However, if there were a systematic bias in the dataset, then there would less likely be a significant statistical relationship between temperature and flowering time. Also, there are many biological factors that may lead to a lack of correlation between these phenophases. First, there is some evidence that there is variation in the response of growing season length to climate change by latitude. Lower latitudes (32° N–37° N) in North America appear to not have an increase in growing season to the same extent as higher latitudes (42° N–45° N)^29^, which could add variance to phenophases, especially on the extremes of life history, such as initial growth and fruit production. Second, initial growth of *A. syriaca* is influenced by photoperiod^30^ in addition to temperature, meaning spring response by *A. syriaca* to increased temperature could be mitigated or at least complicated by physiological responses cued by day length. This could also explain why we found no significant relationship between temperature and initial growth, which could then explain the decoupling of initial growth and flowering. In other words, different environmental factors could be differentially influencing the various phenophases. Third, if phenological mismatches do occur between *A. syriaca* and their pollinators, they could influence fruit production, as well. For example, there is evidence that a majority of fruit in a closely related species (*Asclepias speciosa*) are produced on inflorescences that arise early in the flowering season^31^. If this is also occurring in *A. syriaca* and pollinators are not visiting flowers that are blooming earlier than expected as a result of climate change, then they will not be pollinated, and they will not produce fruit earlier than expected in the fall. Further work must be done to explain the complex interaction of all these factors on the correlation, or lack thereof, between phenophases.

This study is a first step toward understanding the influence of climate change on *A. syriaca* phenology. My results indicate that flowering times have significantly shifted toward earlier in the year as temperatures have increased and this pattern has become more significant from 2011 to 2016. Future work should explore potential relationships between climate change and *A. syriaca*-pollinator interactions with an emphasis on the conservation of this plant, especially in light of its recent decline in many areas^9^ and importance as a host for many insects, including the declining *Danaus plexippus*^13^. In addition, this study illustrates the usefulness of phenological data repositories and citizen science for improving our understanding of the biological consequences of climate change.

## Methods

I used phenological and temperature and precipitation data from the USA-NPN repository for this study^14^. USA-NPN organizes and shares phenological data collected by scientists, citizen scientists, government organizations, non-government organizations, educators, and students that researchers can use to better understand and quantify the biological consequences of climate change. I downloaded all data for *A. syriaca* from the repository for the years 2011 through 2016. I only included “yes” status phenological measurements in our analysis, which indicate the presence of the phenophase, such as flowering. More importantly, I only included “yes” status measurements that were preceded by “no” measurements within a given year to reduce the bias of incomplete surveying that may occur with data collected by citizen scientists. The climate data, daily maximum temperature and precipitation values, for the location of each plant in the USA-NPN repository was provided by DAYMET (https://daymet.ornl.gov), which is a resource provided by NASA that gives daily climate summaries at a 1 km x 1 km spatial resolution. Also, I downloaded monthly state level maximum temperature and precipitation data from the NOAA website^15^ to compare contemporary (2011 through 2016) to historical (dating back to 1895) climate data.

Using these datasets, I quantified the linear relationship between climate (temperature and precipitation) and Julian flowering date for the six-year period, 2011–2016. However, the response of flowering to climate change does not occur in a vacuum. For example, other aspects of plant phenology may also respond to climate change in concert with or opposition to flowering time^32^. This variability in response may be due to the wide array of environmental factors, in addition to temperature and precipitation, which are influenced by climate change and in turn influence many phenological stages, such as germination, vertical growth, and leafing^33^. It is critical to examine many aspects of phenology when quantifying the influence of climate change on a plant’s flowering period. Therefore, to determine if there was any potential influence of the timing of other phenophases on flowering I quantified the linear correlations between flowering and both initial growth and fruit ripening for individual plants within each year. I used initial growth and fruit ripening because they provide full coverage of the annual growth cycle of *A. syriaca*.

I used repeated measures linear regressions to quantify the relationships between mean Julian flowering date of individual plants and mean growing season (March through November) maximum temperature and precipitation, and year. I also included year and temperature and year and precipitation interaction terms to determine if the relationship between flowering and climate is changing through the 6 year time period. I used mean instead of first date values, which are common measures in climate change studies, because first date is an incomplete and extreme measure of a phenophase distribution^34^. However, to further examine the flowering phenophase, I generated models using minimum (first) and maximum (last) flowering dates. The repeated measures portion of the linear regressions controlled for repeated sampling of individual plants during multiple years. The latitudinal range of samples increased significantly from 2011 through 2016. Therefore, I only included samples that fell within the 2011 latitude range. In addition to limiting the latitude range, I also included it in my model along with elevation. All dependent variables, except for the temperature comparison between historical (1895–2010) and contemporary (2011–2016) dates, met the assumptions of parametric analyses. A parametric test is not appropriate for the historical to contemporary temperatures comparison because of the very different sample sizes of the historical versus contemporary data. Therefore, I used a Welch’s t-test. All statistical analyses were carried out using the R Statistical Program^35^. I used the lme4 package^36^ to generated the repeated measures model and the lmerTest package^37^ to run t-tests using the Satterthwaite approximations for degrees of freedom.
